# Predicting Fintech Innovation Adoption: the Mediator Role of Social Norms and Attitudes

**DOI:** 10.1186/s40854-022-00434-6

**Published:** 2023-01-15

**Authors:** A. Irimia-Diéguez, F. Velicia-Martín, M. Aguayo-Camacho

**Affiliations:** 1 Department of Financial Economy and Operations Management, University of Seville, Seville, Spain; 2 Department of Business Management and Marketing, University of Seville, Seville, Spain

**Keywords:** Fintech services, Theory of planned behavior, Theory of reasoned action, PLS-SEM, Predictive modeling, Fintech marketing, G23, G40, M13, M31, O32, O33

## Abstract

Digital innovation is challenging the traditional way of offering financial services to companies; the so-called Fintech phenomenon refers to startups that use the latest technologies to offer innovative financial services. Within the framework of the Theory of Planned Behavior (TPB) and the Theory of Reasoned Action (TRA), the primary purpose of this paper is to develop a causal-predictive analysis of the relationship between Subjective Norms, Attitudes, and Perceived Behavioral Control with the Intention to Use and Behavioral Use of the Fintech services by companies. Partial Least Squares Structural Equation Modeling methodology was used with data collected from a survey of 300 companies. Our findings support the TRA and TPB models and confirm their robustness in predicting companies’ intention and use of Fintech services. Financial technology innovators must understand the processes involved in users’ adoption to design sound strategies that increase the viability of their services. Studying the antecedents of behavioral intention to adopt Fintech services can greatly help understand the pace of adoption, allowing these players to attract and retain customers better. This study contributes to the literature by formulating and validating TPB to predict Fintech adoption, and its findings provide useful information for banks and Fintech companies and lead to an improvement in organizational performance management in formulating marketing strategies.

## Introduction

Digital innovation has burst into finance, generating new business models that disrupt the organizational model of traditional banking. This digital disruption is challenging the traditional way of offering financial services to companies in more agile, flexible, transparent, and economical ways. The Fintech phenomenon comes from the union of the English words Finance and Technology and refers to startups that use the latest technologies to offer innovative financial services. Fintech is a “technologically enabled financial innovation that could result in new business models, applications, processes or products with an associated material effect on financial markets and institutions and the provision of financial services” (EBA 2018). The Fintech sector significantly contributes to the financial system by reducing costs, providing higher-quality services, and increasing customer satisfaction (Kou et al. 2021). To this end, financial providers must develop applications that can automatically detect fraud, predict rejections and assess credit. Thus, it is crucial to understand data patterns that can be used to infer user behavior and identify potential risks (Li et al. 2021).

The use of technologies by financial services firms is not new; financial services have been long implemented by applying internal technological solutions or by relying on outsourcing arrangements with external service providers to provide technological solutions. Nevertheless, in recent years, this process appears to have risen to a new level due to the wide range of financial innovations implemented, the significant investments in new technologies—especially during and after the COVID-19 pandemic—and the blend of new firms entering the financial markets. According to Kou et al. (2021), new studies play an essential role in improving investments in the financial sector, as Fintech is recognized as one of the most important innovations in the financial industry and is evolving rapidly.

Fintech services include various innovative financial services, such as payment technology, crowdfunding platforms, wealth management, insurance, and currencies. Their main features are to enhance customers’ experiences with financial services by increasing transparency, cutting costs, eliminating intermediaries, and making financial information accessible (Shiau et al. 2020). Although Fintech has attracted significant attention, its continuous use is still doubtful, as reports on Fintech services show that potential users may not be using the systems, despite their availability.

Customers’ continued-use intentions have been emphasized as a more critical factor in the success of mobile services than their initial adoption (Zhou et al. 2018). According to Jang et al. (2016), users’ intention does not automatically reflect in users’ behavior; thus, further research is needed to identify the factors determining the intention to use and the users’ acceptance of Fintech services.

The ubiquitous use of Fintech services via mobile technology involves a complex collaboration of technology and human interaction; however, limited research has examined the antecedents of the actual use of the technology (namely, mobile money), and only a few studies have investigated the use of Fintech innovations from both technological and behavioral theory perspectives (Senyo and Osabutey 2020). Thus, our research focuses on the behavioral factors that foster the intention to use and the service’s actual use, with technological factors also included in our research model.

This study aims to identify the factors influencing companies’ intentions toward adopting Fintech services. To this end, our research combines the Theory of Planned Behavior (TPB) developed by Ajzen (1991) and the previous Theory of Reasoned Action (TRA) by Fishbein and Ajzen (1975). From a methodological perspective, our work highlights the importance of assessing a model’s predictive power using the Partial Least Squares (PLS) predictive technique. We conducted a telephone survey of companies and obtained 300 valid results. Our results are critical for financial technology innovators and service providers to understand the processes involved in users’ adoption to design sound strategies that increase the viability of their services.

Our contribution is three-fold. First, this paper combines TPB and TRA to study the influence behind companies’ adoption of Fintech services. These theories have been extensively applied in other fields but not in Fintech services adoption studies. Both theories were selected based on the factors that drive companies to use Fintech services since we consider that the focus must be on accepting the service rather than accepting the technology. Second, from a methodological perspective, there is a need for further quantitative research, including explanatory analyses and predictive studies. Although some studies that apply Partial Least Squares structural equation modeling (PLS-SEM) have stressed the predictive nature of their analyses, the assessment has been based exclusively on techniques designed to evaluate the in-sample predictive power of the models. Therefore, this paper seeks to advance this emerging line of research by testing the model’s out-of-sample predictive power. Third, our research is focused on studying companies’ actual use of Fintech services, a field still unexplored. Organizational acceptance is based on the adoption behavior of the organization’s chief executive officer (CEO) or chief financial officer (CFO), who finally decides whether the company should use new financial services.

The remainder of this paper is organized as follows. In the next section, some relevant literature is reviewed. Then, a conceptual framework and some hypotheses are introduced in detail, along with some reasons for their adoption. The following section introduces the methodology, followed by the data analysis and results. Finally, the discussion and conclusions are presented.

## Literature review and theoretical background

### Theory of Planned Behavior (TPB) and Theory of Reasoned Action (TRA)

The TPB, introduced by Ajzen (1991) as a conceptual extension of the TRA by considering an additional variable, has been proven successful in predicting and explaining human behavior across various information technologies (Ajzen 2002). We aim to contribute to this line of research by theoretically anchoring it in the TPB; thus, we regard Fintech contribution behavior as planned behavior. The assumption is that due to the relative novelty of Fintech services, the friendly interfaces, and their financial implications, companies are not likely to engage in using Fintech services without at least some preliminary considerations.

At its core, the TPB suggests that the likelihood of an individual performing a particular behavior is affected by that individual’s intention to engage in that behavior (Ajzen 1991). According to the extant literature, intentions capture the motivational factors influencing behavior, indicating how hard one is willing to try and how much effort one plans to exert to perform a behavior.

Fishbein and Ajzen (1975) developed TRA based on the idea that any human behavior is affected by a person’s attitude toward that particular action and the outcome that follows. Attitude is a predisposition to interact predictably—favorably or unfavorably—with an object, person, or situation. The influence exerted by other people, known as the subjective norm, represents the perception that a given behavior is more or less expected by the persons considered significant for the decision-maker.

In some cases, considering these two variables has not proven to be sufficient for predicting the behavior of human beings. This assumption is based on TPB, which, as previously indicated, contemplates the concern of a further variable, perceived behavioral control (PBC), to help predict the choices individuals would be willing to make. PBC is the expectation about the ease or difficulty in implementing a certain behavior. In this sense, TPB is proposed to eliminate the limitations of the original model in dealing with behavior regarding which people have incomplete volitional control (Ajzen 1991).

TPB has been widely used to examine the adoption of Internet-based services and Internet-mediated marketplaces by prospective users in many contexts: participation in online communities (Casaló et al. 2010), acceptance of e-services (Hsu and Chiu 2004), adoption of e-commerce (Grandón et al. 2011), adoption of e-banking (Shih and Fang 2004), online trading (Gopi and Ramayah, 2007), online social networking (Baker and White 2010), and spreading of e-WoM (Fu et al. 2015). Nevertheless, few studies investigate its application to Fintech adoption.

By applying TPB, we seek to enhance our understanding of factors contributing to the development of intentions and their behavioral use and complement the limited research on motivational factors in Fintech behavior.

### Behavioral Use (BU) and Intention to Use (BI) Fintech services

This section describes the growing literature on Fintech services. Suryono et al. (2020) stated that the first articles that discuss Fintech were published in early 2014. Before 2014, several articles mentioned digital financial innovations, electronic financial payments, or mobile payments as research for innovations in the financial sector (Gomber et al. 2017). In addition, different studies have focused on a specific type of Fintech, including crowdfunding or entrepreneurial projects. Although scarce, a few broader studies on Fintech exist (Gazel and Schwienbacher 2020).

The main features of the studies about adopting Fintech services included in the Web of Science (WoS) database are shown in Table 1. The WoS examination was performed in January 2021 by searching for “Fintech adoption” and “intention to use Fintech” as keywords in the topic; 102 results were obtained. A filter to select only articles was added, and 78 results were obtained. We read these 78 papers to select only those that analyze the intention to use or use Fintech services. Papers analyzing selected types of Fintech services (Pinochet et al. 2019) were not eliminated. As observed, most studies applied PLS-SEM to determine the factors driving users to adopt different Fintech services.

**Table 1:** Studies on WoS about intention to use Fintech services

Reference	Title	Dependent variable	Independent variables	Theory	Methodology	Data	Country
Chuang et al. (2016)	The Adoption of Fintech Service: TAM perspective	Intention of using Fintech Services in manufacturing industries	Perceived ease of use, attitude, brand and trust	TRA and TAM	PLS-SEM	Survey (440 companies)	Taiwan
Ryu et al. (2018)	What makes users willing or hesitant to use Fintech?: the moderating effect of user type	Fintech continuance intention	Perceived benefit (economic benefit, seamless transaction, convenience), Perceived risk (Financial, legal, security, operational)	TRA	PLS-SEM	Survey (243 respondents with Fintech usage experience)	Korea
Lim et al. (2019)	An empirical study of the impacts of perceived security and knowledge on continuous intention to use mobile Fintech payment services	Continuous intention to use mobile Fintech payment services	Perceived Security, Confirmation, Usefulness, Satisfaction	Extended Post Acceptance Model (EPAM)	PLS-SEM	Survey (149 respondents without Fintech payment experience)	Korea
Hu et al. (2019)	Adoption intention of Fintech services for bank users: An empirical examination with an extended technology acceptance model	Intention to use Fintech Services	Perceived ease of use, Perceived usefulness, Attitude, Brand Image, Trust, Perceived Risk, Government support, User Innovativeness	Extended TAM	PLS-SEM	Survey (387 rural commercial bank users)	China
Pinochet al. (2019)	Propensity of contracting loans services from Fintech’s in Brazil	Propensity to use lending Fintech services	Perceived use, Perceived utility, Personal innovation, Trust, Privacy and Social Influence	Not defined	PLS-SEM	Survey (507 respondents)	Brazil
Wang et al. (2019)	What determines customers´continuance intention of Fintech? Evidence from YuEbao	Intention of Use of YuEbao (a representative example of a Fintech Service in China)	Trust, assurance, technical and social factors	Not Applicable	PLS-SEM	Survey (288 respondents)	China
Jünger and Mietzner (2020)	Banking goes digital: The adoption of Fintech services by German households	Use of Fintech	Trust, Comfort with new technologies, financial literacy and transparency	Not Applicable	Logit Regression and Principal Component Analysis	Survey (323 households)	Germany
Shiau et al (2020)	Understanding Fintech continuance: perspectives from self-efficacy and ECT-IS theories	Fintech Continuance Intention	Financial and Technological Self-Efficacy, Perceived Usefulness, Confirmation and Satisfaction	Self-Efficacy and ECT-IS	PLS-SEM, PLS-predict and multi-group analysis	Survey (753 users with wealth management experience)	China
Singh et al (2020)	What drives Fintech adoption? A multi-method evaluation using an adapted technology acceptance model	Intention of Use and Use of Fintech	Social Influence, Usefulness, Ease of Use, security and Responsiveness	TAM and UTAUT	PLS-SEM and multi-group analysis	Survey (439 internet users)	India

### Theoretical Model and Hypotheses

Based on TPB, the topics listed below are the factors influencing the BU of Fintech services.

### Behavioral Intention to Use (BI)

Fintech companies may not be able to reap the benefits of innovation if the technological advancement is at a higher rate than consumer awareness and use (Abbasi and Weigand 2017). Hence, technology use and adoption have gained the attention of researchers, and several theories and models have been proposed to study the behavioral intention of use. TRA and TPB consider BI the best indicator of BU since it expresses the effort individuals is willing to make to develop a certain action (Ajzen 1991; Armitage and Conner 2001). Thus, our first hypothesis is stated as follows:

#### H1

BI positively affects the BU of Fintech services.

### Subjective Norms (SN)

SN may have an important social influence on the behavior of individuals (Martín-Navarro et al. 2021). When individuals are in groups, there are certain rules, norms, or beliefs about their proper behavior. The group (family, friends, or co-workers) exerts pressure on individuals who feel they may have a certain behavior regarding the reference group (Yasa et al. 2021). In other words, SN is “the individual’s beliefs about whether significant others think he or she should engage in the behavior and are assumed to capture the extent of perceived social pressures exerted on individuals to engage in a certain behavior” (Shneor and Munim 2019).

One way to capture the inferred behavior of others in the current Fintech context may be through comments made by users, experts, and media on Fintech practice and experiences. As such, we propose the following hypotheses:

#### H2

The greater the SN exerted, the greater the BI of Fintech Services.

#### H3

The greater the SN exerted, the greater the positive attitude (AT) toward using Fintech Services.

### Perceived Behavioral Control (PBC)

PBC is “the individual’s perception of how easy or difficult the performance of a certain behavior is, capturing the extent to which he or she views themselves as having the capacity to perform it” (Shneor and Munim 2019). TPB states that PBC is a strong predictor of BU (Baber 2020; Tucker et al. 2019). On this basis, we formulate the following hypotheses:

#### H4

The greater Fintech Services’ PBC, the greater the BU.

#### H5

PBC positively influences AT toward the use of Fintech Services.

### Self-Efficacy (SE)

Companies are more confident if they consider themselves more competent than other firms in performing financial tasks. In this sense, companies judge managers with high financial SE to be capable of controlling and managing the company’s financial situation (Asebedo and Payne 2019). Perceived SE refers to “people’s beliefs about their capabilities to exercise control over their level of functioning and over events that affect their lives” (Bandura 1991; cited in Ajzen 2002). Defined at this general level, perceived SE differs greatly from PBC, which is focused on the ability to perform a particular behavior. SE has been considered one factor that directly or indirectly influences users’ continuance intention in the financial service context (Shiau et al. 2020; Choi 2018).

While the original conceptualization of PBC resembled that of SE (Bandura, 1982), the later literature argued that a dimension capturing one’s belief about the extent to which their efforts can influence the outcome of behavior should be acknowledged and treated separately (Manstead & Eekelen, 1998; Terry and O’Leary 1995). This argument was made by linkage to diverse sources of control, where SE relates to internal controls, such as ability and motivation, while PBC relates to external controls, such as task difficulty, access to resources, securing the cooperation of others, and luck. The argument above is our basis for the following hypothesis:

#### H6

The greater the SE, the greater the PBC.

### Attitude (AT)

Attitude is defined as a personal feeling, either good or bad, regarding accomplishing the intended behavior and how that feeling influences a particular action/object (Fishbein and Ajzen 1975). Many authors (Chong et al. 2021; Ho et al. 2020; Liébana-Cabanillas et al. 2014) state that attitude is vital in influencing an individual to adopt new technology (particularly mobile banking) since it reduces the barrier to adopting innovation and makes transactions feasible. A company’s willingness to use Fintech services depends on how favorably the company views this behavior and has positive expectations about performing it. Positive perspectives can promote one’s intention to contribute and encourage others to contribute by sharing information about the services used. Accordingly, we hypothesize the following:

#### H7

The company’s AT toward using Fintech services implies a greater BI.

**Fig. 1:**
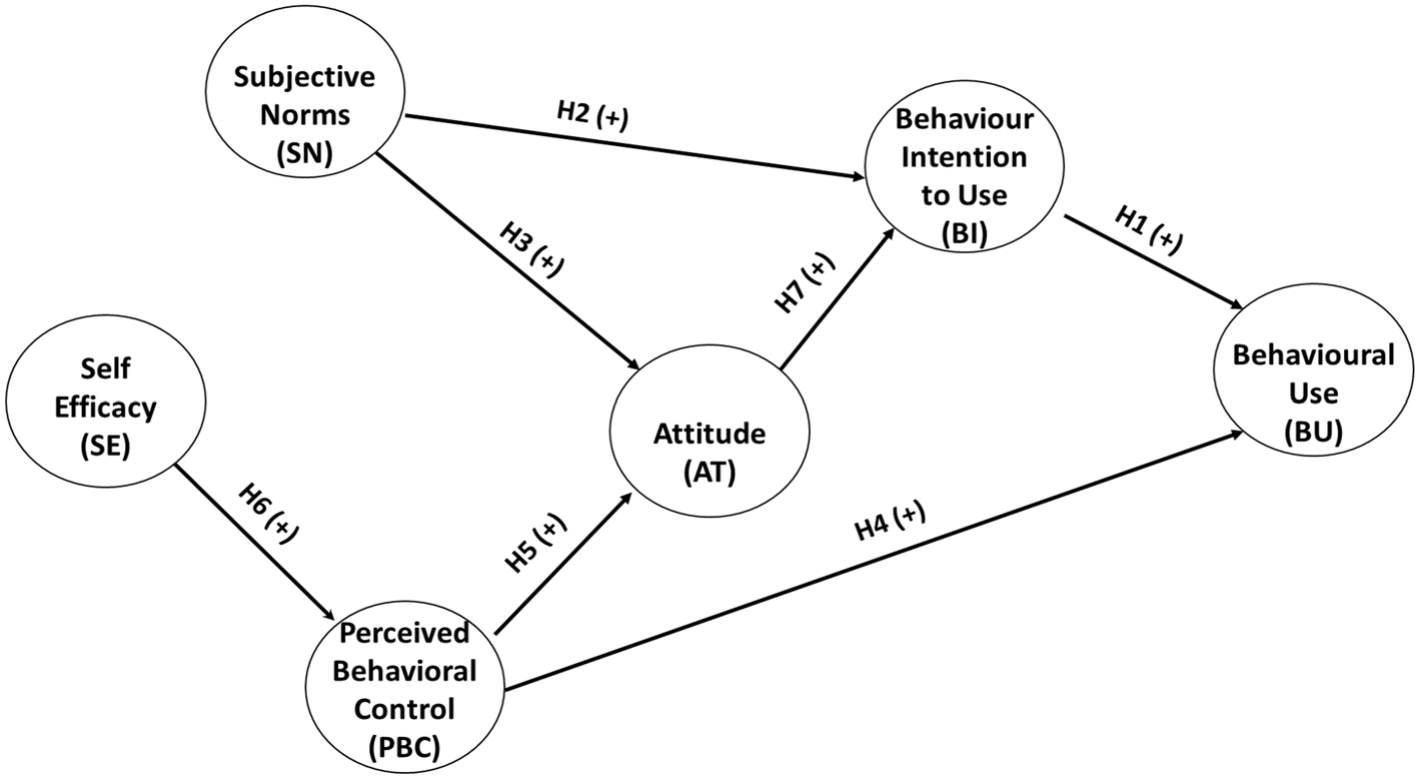
Conceptual model

The abovementioned hypotheses are represented in Fig. 1, which depicts the proposed model.

## Methodology

### Data Collection

A telephone survey was conducted among companies in southern Spain during March and April 2021, obtaining a sample of 300 valid questionnaires. The Andalusian Confederation of Businesses was commissioned to obtain a sample of at least 300 valid responses through the research service of the Andalusian employers’ organization. A pre-test was conducted with 5 experts and 50 participants to ensure the understanding of the questionnaire and its suitability to the objectives of the study.

Non-probabilistic sampling was used. According to Otzen and Manterola (2017), this sampling technique is appropriate when selecting individuals for the study, depending on certain characteristics or criteria fundamental to the research. In our case, individuals who should answer the questionnaire ought to be those with the capacity to make decisions related to financial issues, i.e., either owners or (financial) managers of the company.

In small companies, the CEO carries out the company’s financial management (70.90% of the companies in the sample have 10 or fewer employees, see Table 3), while larger companies usually have a CFO. Thus, the CEO or CFO decides whether to adopt Fintech services; therefore, we focused on their responses.

Information on the purpose of the research was first explained to reduce the dropout rate; the response rate was 78.53% as 382 companies were contacted, and after a filtering process, a valid sample of 300 questionnaires was obtained. Babbie (2007) suggests that a response rate of at least 50% is considered adequate for analysis and reporting, a response of 60% is good, and a response rate of 70% is very good. At the beginning of the data collection process, each respondent was asked to agree to participate voluntarily and was assured that their information would be kept confidential.

We confirm our sample’s suitability by meeting the criteria proposed by Faul et al. (2007) based on statistical power analysis in G*power 3.1.9.2 software. This statistical program was designed to estimate statistical power and effect size (Faul et al. 2009). The minimum sample size required for this study is 146 (where the power level = 0.95, the effect size = 0.15, the significance value = 0.05, and the number of predictors = 6). Therefore, the estimation suggests that our sample size is adequate for this research.

The survey instrument for the current study included measurement scales derived from the literature, as shown in Table 2. Based on the research model, the questionnaire was created and reviewed by experts and researchers for content validity. The questions were formulated using a five-point Likert scale, where one mean strongly disagree, and five means strongly agree.

**Table 2:** Measurement scales

Latent construct	Measurement items	
Attitude (Chuang et al. 2016; Chong et al. 2021; Hu et al. 2019)	AT1:	Fintech Services can facilitate my financial transactions
	AT2	Fintech Services are better than traditional banking services
	AT3	Fintech Services are safe
	AT4	Fintech Services are reliable
Behavioural Intention (Albayati et al. 2020; Al-Saedi et al. 2020; Chong et al. 2021; Ho et al. 2020; Zhong et al. 2021)	BI1	We intend to use Fintech Services in the coming months
	BI2	We think we will use Fintech Services in the coming months
	BI3	We plan to use Fintech Services in the coming months
	BI4	We intend to obtain valuable advantages thanks to Fintech Services in the coming months
Perceived Behavioural Control (Baber 2020; (Chong et al. 2021; Ho et al. 2020; Mazambani and Mutambara 2019; Tucker et al. 2019; Yadav et al. 2015; Zhong et al., 2021)	PBC1	Our company has the necessary resources to use Fintech Services
	PBC2	Our company has the necessary knowledge to use Fintech Services
	PBC3	Fintech Services are compatible with our company's systems (ERP, accountants, etc.)
	PBC4	Our company has a person (or several people) available to give support with the difficulties that may arise
Subjective Norms (Chong et al. 2021; Ho et al. 2020; Mazambani and Mutambara 2019; Tucker et al. 2019)	SN1	My clients and suppliers use Fintech Services
	SN2	Reference companies for us use Fintech Services
	SN3	The companies in our environment that use Fintech Services have more prestige than those that do not use it
	SN4	My competitors use Fintech services
	SN5	The companies in our environment that use Fintech Services are innovative
	SN6	Using Fintech Services is a status symbol in our environment
Behavioural Use (Albayati et al. 2020; Al-Okaily 2020; Malaquias and Silva 2020; Tucker et al. 2019)	BU1	We tend to use Fintech Services frequently
	BU2	We spend a lot of time analysing Fintech Services
	BU3	I get involved with Fintech Services
	BU4	I will recommend using Fintech Services
Self-Efficacy (Al-Saedi et al. 2020; Baber 2020; (Ho et al. 2020)	SE1	Fintech Services would be clear and understandable to the employees of our company
	SE2	It would be easy for our company to become familiar with Fintech services
	SE3	It would be easy for our company to use Fintech services
	SE4	It would be easy for our company to learn how to use Fintech services

Following Armstrong and Overton (1977), a comparative analysis using an independent samples t-test was made between the 25 early and the 25 late respondents on the main study constructs. The independent samples t-test indicated that means differences were insignificant (p < 0.01).

The characteristics of the sample are shown in Table 3. The companies included in the sample represent a wide variety of features. As can be seen in Table 3, different sizes of locations are represented. The number of employees or annual sales volume can measure the company’s size. According to both criteria, the smallest companies represent the highest percentage. Finally, the age and the type of company also show a well-represented variety, and freelance is the most represented type.

**Table 3:** Characteristics of the sample (n = 300) per different criteria

Criteria	Categories	Frequency	%
Size of location	Less than 5000	9	3.01
	From 5001 to 20,000	139	46.49
	From 20,001 to 100,000	51	17.06
	More than 100,000	100	33.44
Number of employees	Until 10	212	70.90
	From 11 to 25	49	16.39
	From 26 to 50	23	7.69
	From 51 to 100	7	2.34
	From 101 to 250	4	1.34
	More than 250	4	1.34
Annual sales	Less than 100 T€	50	16.72
	From 100,001 to 300 T€	155	51.84
	From 300,001 to 1 M€	63	21.07
	From 1001 to 5 M€	19	6.35
	From 5001 to 10 M€	5	1.67
	More than 10 M€	7	2.34
Company age	Less than 1 year	5	1.67
	From 1 to 3 years	16	5.35
	From 3 to 5 years	43	14.38
	From 5 to 10 years	87	29.10
	From 10 to 15 years	59	19.73
	From 15 to 25 years	44	14.72
	More than 25 years	45	15.05
Type of company	Public Limited Company (S.A.)	16	5.35
	Freelance	177	59.20
	Social Economy Enterprises	8	2.68
	Limited Company	98	32.78

All these variables were used as control variables of the dependent variable to assess if a multigroup analysis was necessary. None of the control variables was significant.

### Analysis Method

PLS (Chin 1998; Chin and Newsted 1999; Chin and Todd 1995; Lohmöller 1989) is an approach to Structural Equation Models (SEM). PLS allows researchers to concurrently and simultaneously analyze, combining factor analysis and linear regressions, the relationships between theory-based latent variables and their indicator variables by directly measuring observable indicator variables (Hair et al. 2014). To estimate the model parameters, PLS maximizes the variance explained for endogenous constructs through OLS regressions; in other words, the PLS algorithm aims to minimize the residual variances of the dependent variables (Chin 1998).

Implementing PLS requires following a two-stage procedure (Hair et al. 2014). The measurement model is built in the first stage, which includes the relationships between the latent variables and their indicators. To this end, the scores of the latent constructs are iteratively estimated (the reliability and validity of the measurement model). In the second stage, the final estimates of coefficients (outer weights, loadings, and path coefficients) are calculated using the OLS regressions for each partial regression in the model. The result of this second stage is the structural model, the part of the overall model that proposes relationships between the latent variables (Roldán and Sánchez-Franco 2012). The structural model shows the effects tested with the research hypotheses; consequently, it is the core of the PLS model. The SmartPLS-3 software, developed by Ringle et al. (2015), was used.

## Results

### Measurement (Outer) Model

Measurement model evaluation is the first and essential step in generating PLS results.

According to Roldán et al. (2012), the measurement model for reflective (mode A) constructs is assessed in terms of individual item reliability, construct reliability, convergent validity (Table 4), and discriminant validity (Table 5).

**Table 4:** Composite Reliability and Validity

		Loading	Cronbach’s alpha	rho_A	Composite reliability (CR)	Average variance extracted (AVE)
Attitude (AT)	AT1	0.739	0.891	0.904	0.925	0.757
	AT2	0.864				
	AT3	0.927				
	AT4	0.938				
Behavioural Intention of use (BI)	BI1	0.976	0.980	0.980	0.985	0.943
	BI2	0.984				
	BI3	0.971				
	BI4	0.954				
Perceived Behavioural Control (PBC)	PBC1	0.879	0.846	0.861	0.908	0.768
	PBC2	0.872				
	PBC3 PBC4	0.814 0.738				
Self-Efficacy (SE)	SE1	0.905	0.954	0.956	0.972	0.946
	SE2	0.957				
	SE3	0.945				
	SE4	0.945				
Subjective Norms (SN)	SN1	0.854	0.938	0.942	0.967	0.880
	SN2	0.892				
	SN3	0.884				
	SN4	0.894				
	SN5	0.876				
	SN6	0.836				
Behavioural Use (BU)	BU1	0.905	0.895	0.905	0.928	0.766
	BU2	0.911				
	BU3	0.931				
	BU4	0.740

**Table 5:** Discriminant Validity

	AT	SN	PBC	BI	BU	SE
Attitude (AT)	**0.871**	0.676	0.416	0.630	0.651	0.347
Subjective Norms (SN)	0.622	**0.873**	0.322	0.604	0.607	0.307
Perceived Behavioural Control (PBC)	0.356	0.289	**0.828**	0.157	0.207	0.710
Intention to Use (BI)	0.593	0.585	0.126	**0.971**	0.839	0.074
Behavioural Use (BU)	0.579	0.555	0.144	0.788	**0.875**	0.125
Self-Efficacy (SE)	0.312	0.293	0.642	0.067	0.041	**0.938**

The main diagonal shows in bold the square root of the AVE

The Fornell–Larcker Criterion is shown below the main diagonal, whereas the HTMT is presented above the main diagonal

First, individual item reliability is considered valid when an item has a factor loading greater than 0.7, implying that the shared variance between the construct and its indicators is greater than the error variance (Carmines and Zeller 1979).

Second, the construct reliability is assessed using a measure of internal consistency: composite reliability (CR). Following Nunnally (1994), values, which are greater than 0.8, are required for basic research.

Third, the average variance extracted (AVE) measure is applied to assess each construct’s convergent validity. AVE measures the percentage of the variance of a construct explained by its indicators, and these values should be greater than 0.50 (Fornell and Larcker 1981). Since all the constructs are valid with the previously defined criteria, our model is correctly specified from a theoretical point of view.

Assessing the discriminant validity of the constructs is also necessary; therefore, we use the traditional Fornell–Larcker criterion and the Heterotrait–Monotrait ratio of correlations (HTMT) even though Henseler et al. (2015) suggest that HTMT is theoretically superior to the Fornell–Larcker criterion since HTMT achieves high specificity and sensitivity rates across all simulation conditions. Under the Fornell–Larcker framework, the AVE should be greater than the variance shared between the construct and other constructs in the model. Roldán and Sánchez-Franco (2012) and Roldán et al. (2012) suggest that the diagonal elements should be significantly greater than the off-diagonal elements in the corresponding rows and columns to obtain adequate discriminant validity. Table 5 shows that this condition is satisfied for all the constructs of the resulting model. In addition, all the constructs have an HTMT value lower than 0.85, indicating the existence of discriminant validity for all the constructs.

It is also essential to analyze the possibility of Common Method Bias (CMB) and multicollinearity between predictive variables. CMB occurs when variations in responses are caused by the instrument rather than the respondents’ actual predispositions that the instrument attempts to uncover. A comprehensive collinearity test introduced by Kock and Lynn (2012) is recommended for assessing the presence of collinearity and the possible CMB. As Table 6 shows, all inner VIFs among the latent constructs range from 1.000 to 1.631, lower than the threshold of 3.3 proposed by Kock (2015); therefore, the full collinearity test resulted in satisfactory VIFs, and the model is considered free of CMB.

**Table 6:** Multicollinearity test

Inner VIF values	AT	BI	BU	PBC
Attitude (AT)		1.631		
Behavioural Intention to Use (BI)			1.016	
Behavioural Use (BU)				
Perceived Behavioural Control (PBC)	1.091		1.016	
Self-Efficacy (SE)				1
Subjective Norms (SN)	1.091	1.631		

### Structural (Inner) Model

After analyzing the measurement model and confirming that the measurement in the model met the conventional standards of reliability and validity, the next step in the PLS analysis is to evaluate the structural model and test the proposed hypotheses.

The evaluation of the structural model is based on the sign, magnitude, and significance of the structural path coefficients and the R^2^ values (Roldán and Sánchez-Franco 2012). Additionally, as recommended by Henseler et al. (2015), we also report the standardized root mean square residual (SRMR). SRMR has a value of 0.070 as an approximate measure of the overall model fit, which met the cut-off value suggested by Hu and Bentler (1999). We ascertain the statistical significance of the path coefficients by performing a bootstrapping procedure with 5000 resamples (Hair et al. 2011). This approach allows standard errors and generates the t-statistics; however, a percentile bootstrap 95% confidence interval is also employed, which has the advantage of being completely distribution-free (Chin 2010). Following the recommendations of Williams and MacKinnon (2008), the significance of all mediating relations is only tested by using the percentile bootstrap 95% confidence interval. This approach considers that the indirect effect is significantly different from 0 with a 95% confidence level when the interval for a mediation hypothesis does not contain zero.

The results support most of the hypotheses proposed. As observed in Table 7, the variable with the highest impact on BU is BI, according to TPB and TRA. SN and AT have the most potent effect on the intention to use Fintech services.

**Table 7:** Significant testing results of the structural model path coefficients

Hyp.	β (Standard Path Coeff.)	T Statistics	P Values	CI	Sig	Decision
H1: BIBU	0.782	27.898	0.000	(0.734;0.828)	Yes	Supported***
H2: SNBI	0.352	4.677	0.000	(0.228;0.473)	Yes	Supported***
H3: SN AT	0.567	12.011	0.000	(0.489;0.643)	Yes	Supported***
H4: PBCBU	0.046	1.136	0.128	(0.021;0.119)	No	Not supported
H5: PBCAT	0.192	2.428	0.008	(0.057;0.314)	Yes	Supported**
H6: SEPCB	0.642	12.839	0.000	(0.557;0.721)	Yes	Supported***
H7: ATBI	0.374	4.734	0.000	(0.243;0.502)	Yes	Supported***

Significant ( +) Positive relationship: Significant at p*** =  < 0.001, p** < 0.05, p* < 0.1, and No sig. > 0.10

Concerning the coefficient of determination (R^2^), the R-squared value represents the proportion of variation in the endogenous latent variables that can be explained by the effect of one or more exogenous latent variables. The judgment of the R^2^ value is highly dependent on the specific research discipline. Accordingly, some researchers, such as Hair et al. (2014), suggested that the R^2^ above 0.67 can be considered high, while values ranging from 0.67 to 0.33 are moderate, and values between 0.33 and 0.19 are weak; any R^2^ values less than 0.19 are unacceptable.

**Fig. 2:**
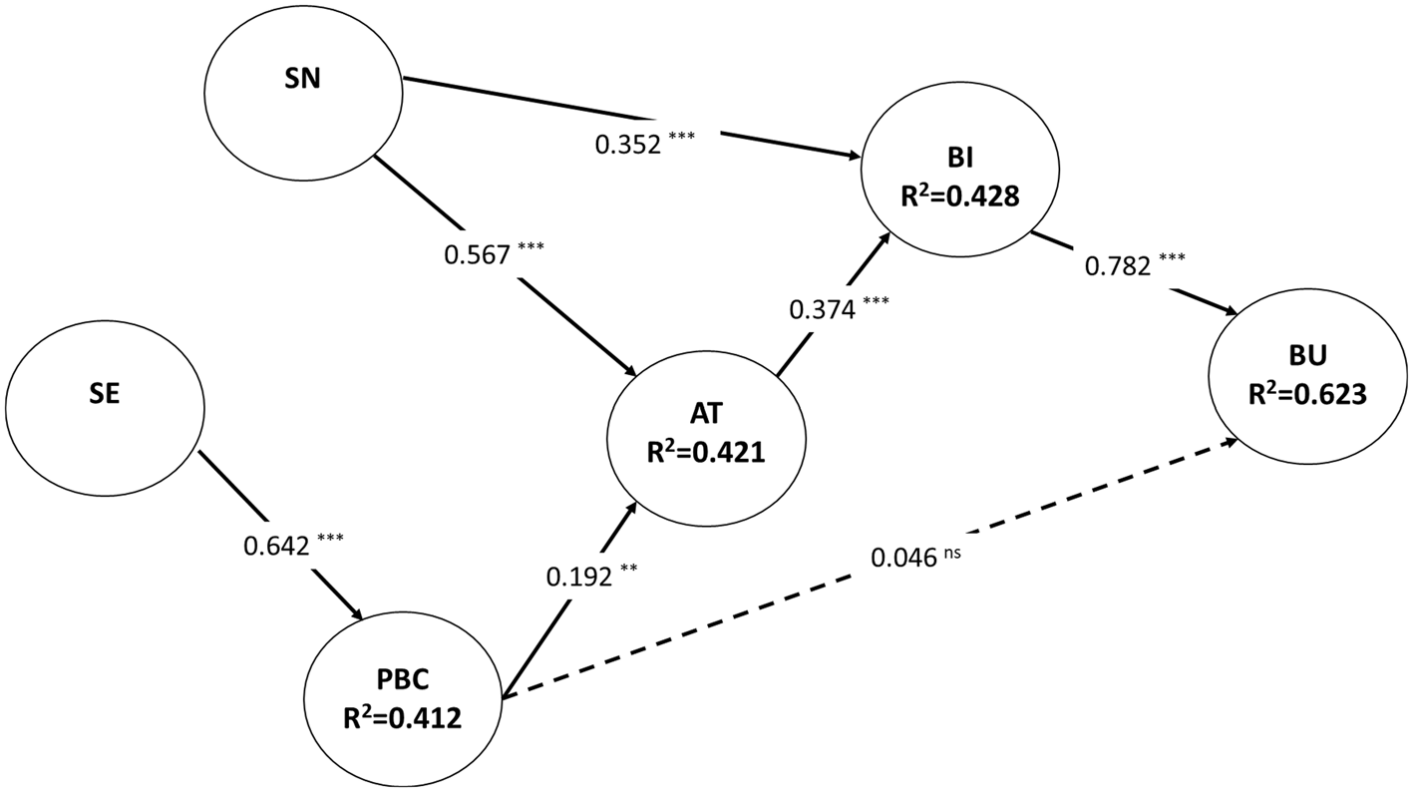
PLS results for the entire sample

Based on the results reported in Fig. 2, the model explains 62.3% of the variance of BU of Fintech services, which can be considered high. In addition, the intermediate variances can be considered moderate.

Our results show that all the variables of TPB (AT, SN, and PBC) significantly influence the company’s intention to use Fintech services (BI), which matches the TPB model. Furthermore, the findings also reported that only two TPB variables (AT and SN) significantly influence the company’s BU, while PBC does not directly influence BU. Nevertheless, the indirect effect of PBC on BU via AT and BI must be considered.

Furthermore, the research model contains other indirect effects that should be analyzed using a bootstrap method with n = 10,000 (see Table 8).

**Table 8:** Summary of Mediating Effects

	Path			*p *value		
*Total effects*						
Total effect of SN on BI	0.564			0.000		
Total effect of PBC on BU	0.046					
*Direct effects*						
H2 ( +): SN on BI	0.352			0.000		
H4 ( +): PBC on BU	0.046			0.128		

	Path	p value	CI (5.0%)	CI (95.0%)	Sig	VAF (%)
*Indirect effect of SN on BI via AT*						
H3 ( +): SN on AT	0.567	0.000	0.478	0.634		
H7 ( +): AT on BI	0.374	0.000	0.233	0.493		
Total SN–AT–BI	**0.212**	**0.000**			**Yes**	**37.7**
H5 ( +): PBC–AT	0.192	0.008	0.054	0.312		
H7 ( +): AT–BI	0.374	0.000	0.233	0.493		
H1 ( +): PBC–BU	0.782	0.000	0.731	0.824		
Total PBC–AT–BI–BU	**0.056**	**0.000**			**Yes**	**54.9**

**Fig. 3:**
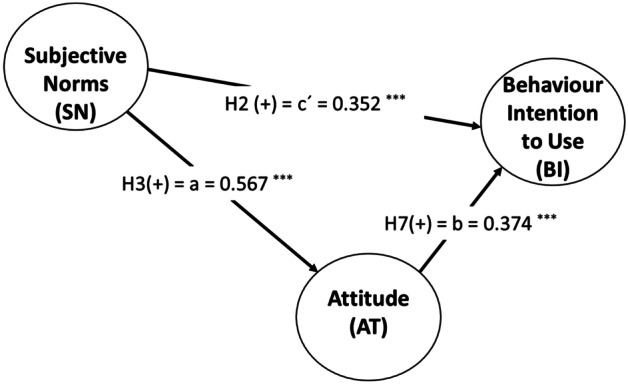
Partial mediation

First, AT mediates the effect of SN on BI. Nitzl et al. (2016) found a partial mediation effect since both the indirect and the direct effects are significant. SN has a significant total effect on BI (c = 0.564, *p*-value = 0.000) due to the significance of the indirect effect via AT (*axb* = 0.212, *p*-value = 0.000) and the direct effect (c’ = 0.352, *p*-value = 0.000). Since the sign of the direct and indirect effects are both positive, the partial mediation effect is considered complementary. One approach to have further information of the mediated portion is calculating the ratio of the indirect-to-total effect also known as Variance Accounted For (VAF) value. A VAF value higher than 80% represents full mediation, a VAF value between 20% and 80% means a partial mediation, while a value below 20% means no mediation. Thus, the result (VAF = 37.66%) demonstrates that AT partially mediates the influence of SN on BU (Fig. 3).

**Fig. 4:**
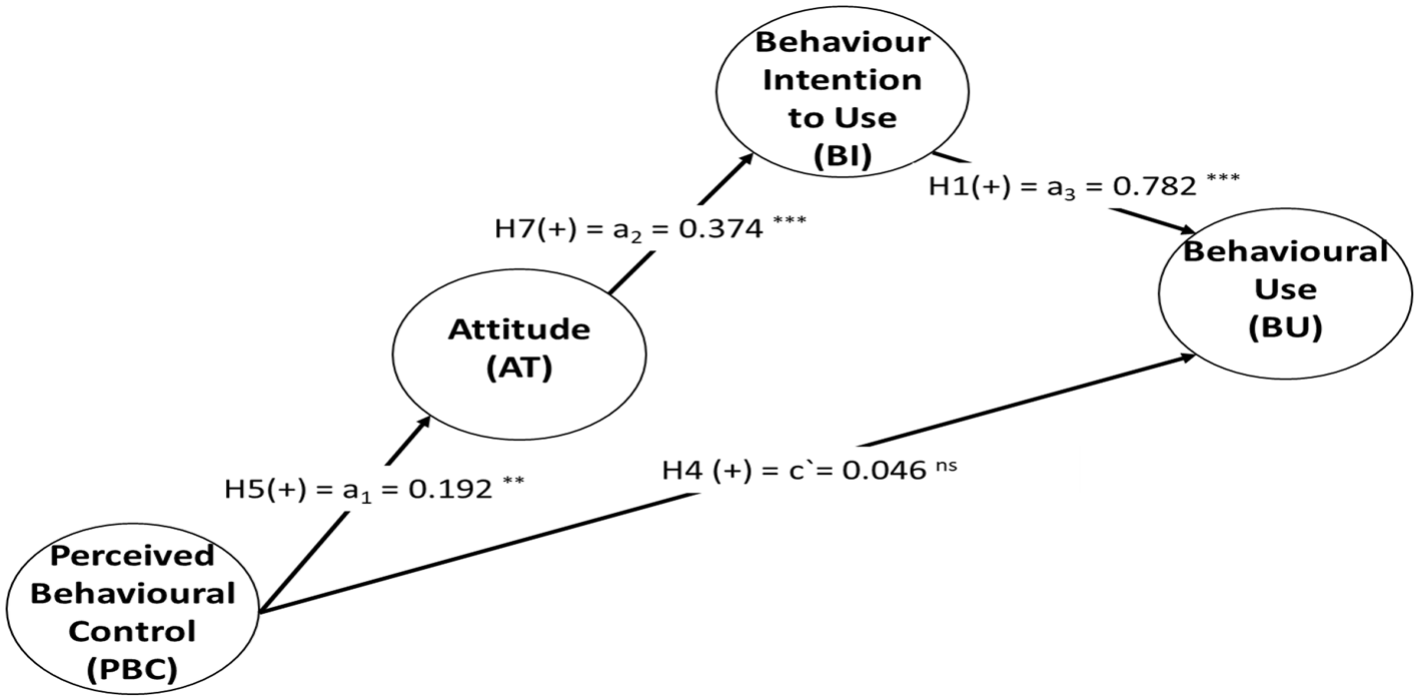
Full mediation

Second, both AT and BI mediate the effect of PBC on BU. According to Nitzl et al. (2016), a full mediation effect is indicated when the direct effect is not significant, whereas the indirect effect is significant. As shown in Fig. 4, PBC has a significant total effect on BI (c = 0.102, *p* value = 0.000) due to the significance of the indirect effect via AT and BI (*axb* = 0.056, *p* value = 0.000) and the direct effect (c’ = 0.046, *p* value = 0.000). The strength of this complete mediation can be assessed by calculating VAF = 54.90%. This result demonstrates that AT and BI fully mediates the influence of PBC on BU.

### Testing the Model’s Predictive Power

This study further applied PLS_predict_ to determine the predictive power of the proposed model. Most research interprets the coefficient of determination (R^2^), which assesses the in-sample model fit of the dependent constructors’ composite scores by using the model estimates to predict the case values of the total sample. Still, the R^2^ value only assesses a model’s explanatory power and provides no indication of its out-of-sample predictive power in the sense of its ability to predict the values of new cases not included in the estimation process (Shmueli et al. 2016).

The model’s out-of-sample predictive power allows testing the model’s generalizability to other populations, which was performed by applying PLS_predict_, a hold-out sample-based approach developed by Shmueli et al. (2016) and recently applied by Calvo-Mora et al. (2020) and Shiau et al. (2020). In the PLS_predict_ routine, first, k-fold cross-validation was carried out, setting k = 10 subgroups to meet the minimum size of N = 30 for the hold-out sample, repeating this process 10 times (Hair et al. 2020). Second, a PLS_predict_ analysis was conducted by completing the following steps.

1. The indicators of the four critical endogenous constructs show Q^2^_predict_ > 0, suggesting that all the manifest variables meet the first requirement. Furthermore, Table 9 shows that since most of the values of Q^2^_predict_ are between 0.25 and 0.5, the predictive relevance is medium (Hair et al. 2019).
2. The values of prediction error summary statistics were compared to naive values obtained by a linear regression model (LM) to evaluate the prediction error of the PLS-SEM analyses (Danks and Ray 2018; Shmueli et al. 2019). Compared to the LM results, the PLS-SEM results should have a lower (negative sign) predictive error in terms of root mean squared error (RMSE) when the prediction errors are highly symmetrically distributed or, otherwise, in terms of mean absolute error (MAE) values (Hair et al. 2019). The last column of Table 9 shows that since not all the values of the skewness for prediction errors of results indicators are under/1/, both RMSE and MAE were selected as a basis of the predictive power assessment.

**Table 9:** PLS_predict_ assessment of indicators

	PLS			LM			PLS–LM		Skewness Predic. errors
	RMSE	MAE	Q^2^_predict	RMSE	MAE	Q^2^_predict	RMSE	MAE	
AT4	0.604	0.459	0.358	0.630	0.468	0.302	− 0.026	− 0.009	0.7270
AT2	0.692	0.527	0.279	0.722	0.533	0.216	− 0.030	− 0.006	0.3040
AT3	0.649	0.483	0.314	0.670	0.493	0.269	− 0.021	− 0.010	0.9290
AT1	0.692	0.484	0.226	0.690	0.494	0.232	0.002	− 0.010	− 0.7300
PBC3	0.670	0.536	0.163	0.676	0.531	0.151	− 0.006	0.005	− 0.1670
PBC4	0.720	0.505	0.268	0.760	0.527	0.185	− 0.040	− 0.022	− 1.4830
PBC2	0.693	0.546	0.269	0.712	0.552	0.227	− 0.019	− 0.006	− 0.5900
PBC1	0.626	0.417	0.376	0.642	0.430	0.345	− 0.016	− 0.013	− 1.1320
BI1	0.741	0.594	0.328	0.728	0.566	0.352	0.013	0.028	1.1420
BI2	0.760	0.618	0.290	0.741	0.585	0.325	0.019	0.033	1.0040
BI4	0.691	0.549	0.325	0.685	0.531	0.338	0.006	0.018	1.1640
BI3	0.762	0.589	0.262	0.759	0.566	0.267	0.003	0.023	1.2940
AU1	0.875	0.686	0.175	0.884	0.646	0.195	− 0.009	0.040	0.8500
AU2	0.786	0.544	0.183	0.781	0.552	0.194	0.005	− 0.008	1.1960
AU3	0.853	0.659	0.216	0.868	0.657	0.188	− 0.015	0.002	0.7280
AU4	0.850	0.707	0.247	0.864	0.685	0.223	− 0.014	0.022	0.2350

Table 9 also shows that PLS-SEM analysis generated lower RMSE prediction errors (when the prediction errors are highly symmetrically distributed) for most indicators than the LM estimates. Thus, it can be maintained that the model has a medium predictive power.

The structural analysis supports most of the hypotheses analyzed with the β of each construct. In this vein, H1 is supported by a 99.9% confidence level, indicating that BI positively affects BU. H2 was also supported with a 99.9% confidence level; the greater SN exerted, the greater BI of Fintech services. The same applies to H3; the greater SN exerted, the greater the positive AT toward Fintech services. Moreover, H7 was also supported with a 99.9% confidence level, meaning that the company’s AT toward using Fintech services implies a higher BI.

The relationship between SN, the company’s AT, and BI also shows significant indirect effects. In this sense, AT mediates the effect of SN on BI, as shown in Fig. 3. SN has a significant total effect on BI (c = 0.564, *p-value* = 0.000) due to the significance of the indirect effect via AT (a *x* b = 0.212, *p value* = 0.000) and the direct effect (c’ = 0.352, *p*-value = 0.000). This result demonstrates that AT partially mediates the influence of SN on BU.

Finally, H6 is supported at a 99.9% confidence level, showing that the greater SE, the greater PBC. A company’s confidence is higher when it has greater competence in controlling and managing financial tasks (Asebedo & Payne, 2019).

On the contrary, H4 is not initially supported, implying that a greater PBC of Fintech services does not mean a higher BU.

## Discussion

This research focuses on a highly topical issue: users’ acceptance of Fintech services. The results of this study show that TPB perfectly applies to study the adoption of Fintech services by small and medium enterprises (SMEs) since our model explains 62.3% of the variance of the BU of Fintech services, which can be considered satisfactory. Furthermore, our model shows an adequate out-of-sample predictive power of the model, which allows testing the generalizability of the model to other populations.

Our results show, first, the variable which has the highest impact on the use of Fintech services is the intention to use, in line with Singh et al. (2020). In other words, the intention to use Fintech services is a good predictor of usage behavior. Second, SN and AT have the strongest effect on the intention to use Fintech services. The positive relationship between SN and BI is also supported by Pinochet et al. (2019) or Wang et al. (2019) in the Fintech sector, as well as Al-Okaily et al. (2020) or Al-Saedi et al. (2020) in digital payments vertical. Notwithstanding, Singh et al. (2020) found a negative relationship between SN and BI; thus, on the one hand, the information that users share about their experiences with Fintech services can influence the intention of other users. On the other hand, attitude (understood as a favorable or unfavorable individual sentiment toward an action) is confirmed as a crucial element in adopting Fintech services and is formed based on social influence. These findings imply that one decisive element par excellence is the establishment of the intention to use Fintech services in a virtual environment. The formation of positive attitudes toward Fintech services among potential customers can increase their intention to adopt them.

Thirdly, the abovementioned relationship is more significant than PBC, which may be due to the high-friendly apps offered by Fintech companies that focus their strategy on the User Experience. Thus, companies’ motivation to use Fintech services is based more on the image and reputation of their customers, providers of services, and competitors than on the resources available to use Fintech services.

Conversely, since the relationship between PBC and BU is significant only mediated by AT and BI, our results show that the use of Fintech services does not depend on the user perception of the difficulty level of the Fintech service. Thus, technology (namely, the use of apps or web pages) does not seem to be an obstacle to adopting Fintech because the technological base is not unknown from the user’s perspective. Considering that Fintech services are based on friendly and easy-to-use apps, this fact may be crucial to decide the driver of using Fintech services. Indeed, the fear seems to arise from the scarce knowledge that those in charge of SMEs have of the new financial services. This fact is highly relevant for those companies that provide these Fintech services since their plans should focus on developing word-of-mouth strategies and obtaining current clients to recommend the service directly.

An additional strategy for Fintech companies could be attempting to establish these services as prestigious or as a status symbol. Messages showing that Fintech users are prestigious or innovative companies could be suitable for accepting these services by late adopters.

Our research aims to reduce the gap in empirical works related to accepting Fintech services. It also provides suggestions to managers of both financial institutions and Fintech companies and developers of new financial services linked to technologies to design sound strategies that increase the viability of their services and attract companies to Fintech services.

### Theoretical implications

Our study makes several contributions to the research on adopting Fintech services. First, our work is one of the first to propose and empirically evaluate a behavioral and technological model that combines TPB and TRA. Consequently, this improves scientific knowledge and the academic literature on consumer behavior.

Second, our results show that satisfaction with traditional banking services is highly compatible with Fintech services. This conclusion, obtained from the survey, highlights the competitiveness and the complementarity of the services offered. Most of our respondents are freelancers or companies with less than ten employees, meaning that using Fintech services requires less staff.

Third, this study improves the understanding of the effects of SN on the AT and intention to use Fintech services through the analysis of partial mediation. The present study shows that both characteristics had a considerable influence of similar intensity. In addition, a full mediation of PBC via AT and BI on BU is also shown; thus, this research helps improve the understanding of these variables’ importance by analyzing their mediated effects within the Fintech sector.

Fourth, our results demonstrate the importance of intention to use as the main antecedent of BU. This relationship corroborates the importance of this variable in general innovations, but more specifically in innovations related to financial technologies.

Fifth, the proposed model shows a high-significant relationship between SE and PBC, extending previous studies’ results to the Fintech context.

Finally, the out-of-sample predictive power of the model proposed was tested by applying PLSpredict, which provides the ability to predict the values of new cases not included in the estimation process. Most research interprets the coefficient of determination, which assesses the in-sample model fit of the dependent constructors’ composite scores to the novelty of the Fintech sector; thus, the model’s predictive power becomes even more important.

### Managerial implications

This research also has important implications for providers offering these financial services. First, Fintech companies need to improve usability among users, and, to this end, companies may be one of their main targets due to the possible influence on their employees related to the use of these innovative financial services.

Second, to improve user confidence, service providers should pay attention to SN, including word-of-mouth strategies in their action plans, and reward current clients for recommending the service directly.

Finally, the Fintech sector should consider the changes in consumer behavior due to social changes in the face of the COVID-19 pandemic (Liébana-Cabanillas et al. 2022). In recent months, new Fintech services have been established to reduce the contact between buyer and seller.

### Limitations and Future Lines of Research

One of the study’s main limitations is that the sample is made up of potential Spanish companies. Future research could include users from other countries to generalize the theoretical conclusions reached in this study. Another limitation is the type of online survey based on a structured questionnaire to determine the recommendation intention of the surveyed users at a given time. Future research could use longitudinal data and a mixed-method approach with qualitative techniques such as fs-QCA.

Finally, the authors of this study also suggest that future studies may be conducted to explore the role of personality traits in technological behavior. Liu et al. (2021) suggest that personality traits can be meaningful in explaining individual heterogeneity in household energy-saving behavior, while Li et al. (2022) reveal the critical impact of personality traits on in-hotel pro-environmental behaviors.

## Data Availability

The datasets used during the current study are available from the corresponding author on reasonable request.

## Abbreviations

AT: Attitude
BU: Behavioural use
BI: Intention to use
PLS-SEM: Partial least squares structural equation modeling
PBC: Perceived behavioural control
SE: Self-efficacy
SN: Subjective norms
TPB: Theory of planned behaviour
TRA: Theory of reasoned action
WoS: Web of Science
